# A data processing pipeline for the AACR project GENIE biopharma collaborative data with the {genieBPC} R package

**DOI:** 10.1093/bioinformatics/btac796

**Published:** 2022-12-15

**Authors:** Jessica A Lavery, Samantha Brown, Michael A Curry, Axel Martin, Daniel D Sjoberg, Karissa Whiting

**Affiliations:** Department of Epidemiology and Biostatistics, Memorial Sloan Kettering Cancer Center, 633 3rd Avenue New York, New York 10017, United States; Department of Epidemiology and Biostatistics, Memorial Sloan Kettering Cancer Center, 633 3rd Avenue New York, New York 10017, United States; Department of Epidemiology and Biostatistics, Memorial Sloan Kettering Cancer Center, 633 3rd Avenue New York, New York 10017, United States; Department of Epidemiology and Biostatistics, Memorial Sloan Kettering Cancer Center, 633 3rd Avenue New York, New York 10017, United States; Department of Epidemiology and Biostatistics, Memorial Sloan Kettering Cancer Center, 633 3rd Avenue New York, New York 10017, United States; Department of Epidemiology and Biostatistics, Memorial Sloan Kettering Cancer Center, 633 3rd Avenue New York, New York 10017, United States

## Abstract

**Motivation:**

Data from the American Association for Cancer Research Project Genomics Evidence Neoplasia Information Exchange Biopharma Collaborative (GENIE BPC) represent comprehensive clinical data linked to high-throughput sequencing data, providing a multi-institution, pan-cancer, publicly available data repository. GENIE BPC data provide detailed demographic, clinical, treatment, genomic and outcome data for patients with cancer. These data result in a unique observational database of molecularly characterized tumors with comprehensive clinical annotation that can be used for health outcomes and precision medicine research in oncology. Due to the inherently complex structure of the multiple phenomic and genomic datasets, the use of these data requires a robust process for data integration and preparation in order to build analytic models.

**Results:**

We present the {genieBPC} package, a user-friendly data processing pipeline to facilitate the creation of analytic cohorts from the GENIE BPC data that are ready for clinico-genomic modeling and analyses.

**Availability and implementation:**

{genieBPC} is available on CRAN and GitHub.

## 1 Introduction

Data abstraction from electronic health records (EHR) has led to a surge in the number of large databases available for analyses of oncologic outcomes. These databases allow for in-depth assessment of the effectiveness of new treatments or risk stratification based on genomic characterization. One such database is from the American Association for Cancer Research Project Genomics Evidence Neoplasia Information Exchange Biopharma Collaborative (GENIE BPC), which represents a multi-institution coordinated effort to aggregate clinical and genomic data for approximately 15 000 patients with cancer over the course of 5 years (Lavery *et al.*, 2022; Litchfield *et al.*, 2017; Micheel *et al.*, 2018).

Project GENIE BPC is a data curation effort to augment the existing genomic data from Project GENIE (AACR Project GENIE Consortium, 2017) with phenomic data to create a comprehensive clinico-genomic data repository. Curated phenomic data include detailed information on cancer diagnosis, drug regimens, disease status from radiology reports, pathology reports and medical oncologist assessments, structured in multiple phenomic datasets with over 700 feature variables (Schrag, 2018). Integrating genomic data from Project GENIE with phenomic data from Project GENIE BPC to produce a record of therapeutic, clinical and genomic information can be used to advance translational research. Analyses of linked clinico-genomic data such as that of GENIE BPC are essential to propelling the precision medicine aim of discovering which treatment interventions and genetic features yield the best clinical outcomes.

The GENIE BPC data are publicly released by cancer cohort using the data-sharing platform Synapse (Sage Bionetworks, 2022). The data are stored as comma-separated value (.csv) and text (.txt) files alongside the Analytic Data Guide documentating each data file, its structure, and the variables included. Data are made accessible for users to download through the Synapse platform. However, accessing the data in this fashion is inefficient for establishing fully reproducible data analysis workflows contained within R programming environments. New versions of the data are also released at regular intervals and may include data corrections as a result of ongoing quality assurance processes, additional variables, and additional curated cases, emphasizing the need for version control regarding the data release used for each analysis.

The {genieBPC} package is a user-friendly R package that establishes a reproducible data processing pipeline that can be used to obtain the data corresponding to any GENIE BPC data release available on Synapse and to create clinical and genomic data frames that are structured for analysis in the R programming language. This data processing pipeline circumvents the need to manually download the data by providing tools to programmatically pull the data into an R environment, allowing users to integrate this step into their reproducible and version-controlled analysis scripts. Further, {genieBPC} enables building analytic cohorts based on streamlined functions with criteria related to diagnosis and treatment, providing readable code that appropriately accounts for the complex underlying data structure.

We present a case study to demonstrate the workflow for using the {genieBPC} R package to download the GENIE BPC data and create an analytic cohort of patients with stage IV non-small cell lung cancer (NSCLC) for statistical analyses. The {genieBPC} R package and additional technical details are available on GitHub and CRAN. The most recent version of the package can be installed using ***install.packages(‘genieBPC’)*** and ***library(genieBPC)*** to load the package. At the time of publication, the version of {genieBPC} available on CRAN was 1.0.1. The code ***remotes::install_github(‘GENIE-BPC/genieBPC’)*** can be used to install the development version from GitHub. The R package includes formal tests using the {testthat} package, as well as testing on Windows, Linux and Unix systems using continuous integration via GitHub Actions.

## 2 {genieBPC} case study

**Fig. 1.**
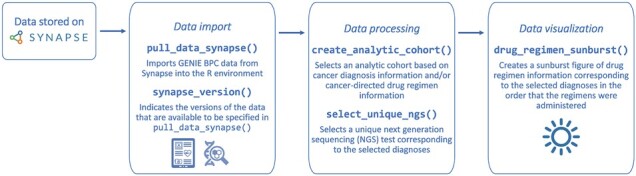
Data processing pipeline for GENIE BPC data using the {genieBPC} package

The case study demonstrates the creation of an analytic cohort of patients diagnosed with Stage IV adenocarcinoma NSCLC who were treated with Carboplatin or Cisplatin and Pemetrexed with and without Bevacizumab as their first cancer-directed drug regimen. This example details the GENIE BPC data processing pipeline using the ***pull_data_synapse(), create_analytic_cohort()***and***select_unique_ngs()***functions (Fig. 1). After running the three functions, the user will have a dataset that is ready to undergo clinico-genomic analyses and that can be visualized in an interactive sunburst plot using the ***drug_regimen_sunburst()*** function.

The data used for this case study are publicly available and are hosted on Synapse.

### 2.1 Read data from Synapse into the R environment: *pull_data_synapse()*

In order to pull the GENIE BPC data from where they are stored on the Synapse platform into the R environment, a user must create a Synapse account and store their Synapse credentials in the R environment. The ***set_synapse_credentials()*** function can be used to set Synapse credentials during each R session:

set_synapse_credentials(username = ‘your_username’,

             password = ‘your_password’)

To avoid having to enter authentication credentials in each R session, Synapse credentials can also be stored in the .*Renviron* file. To do so, a user can add the following to the .*Renviron* file (use ***usethis::edit_r_environ()*** to easily open/edit this file), then restart the R session:

SYNAPSE_USERNAME = <your-username>

SYNAPSE_PASSWORD = <your-password>

Once credentials to download the data are stored in the R environment, the user may proceed to viewing the GENIE BPC data releases that are currently available for download. The ***synapse_version()*** function is a helper function that returns a data frame indicating which cohorts and data release versions are currently available. The code ***synapse_version(most_recent = TRUE)*** will return the list of the most recently available data (Table 1).

**Table 1.** Most recently released versions of the data for each cancer cohort returned by the ***synapse_version()*** function

Cohort	Version	Release_date	Versions_returned
**BrCa**	v1.1-consortium	October 2021	Most recent versions
**CRC**	v1.2-consortium	August 2021	Most recent versions
**NSCLC**	v2.1-consortium	August 2021	Most recent versions
**NSCLC**	v2.0-public	May 2022	Most recent versions

*Note*: As of version 1.0.1 of {genieBPC}.

Setting most_recent = FALSE, FALSE will return a list of all available versions of the data; it is recommended that the most recent versions be used for analyses; BrCa, breast cancer; CRC, colorectal cancer; NSCLC, non-small cell lung cancer.

Each data file on Synapse has an associated Terms of Use regarding data identification, data sharing and publication. The data Terms of Use must be accepted before accessing the data using the ***pull_data_synapse()*** function. Prior to pulling a data release from Synapse into R for the first time, navigate to the Synapse page for the data release and accept the Terms of Use (e.g. for the NSCLC 2.0-public data release, navigate to the Synapse page for the data release). The Terms of Use can be found towards the top of the page, where there is information including the *Synapse ID*, *Item count* and *Access*. Next to *Access* is a link that reads *Request Access*. Select *Request Access*, review the Terms of Use and select *Accept*. Note that these Terms of Use are distinct from the Terms of Use accepted when creating a Synapse account.

The ***pull_data_synapse()***function can then be used to pull the NSCLC clinical and genomic data corresponding to release version 2.0-public from Synapse into the R environment:

nsclc_cohort <- pull_data_synapse(cohort = ‘NSCLC’,

version = ‘v2.0-public’)

This function call returns the object *nsclc_cohort$NSCLC_v2.0*, which includes a nested list of the data frames listed in Table 2.

**Table 2.** Data frames returned by ***pull_data_synapse()***

Data (naming convention)	Description	Structure
**Clinical Data**		
**Patient characteristics (pt_char)**	Patient characteristics and vital status	One record per patient
**Cancer diagnosis (ca_dx_index and ca_dx_non_index)**	Cancer diagnosis characteristics	One record per cancer diagnosis per patient. Index cancers are cancers with associated genomic sequencing.
**Cancer-directed drug regimens (ca_drugs)**	Drug regimens corresponding to each cancer diagnosis	One record per drug regimen and its associated cancer diagnosis, per patient.
**Pathology (prissmm_pathology)**	All pathology reports following cancer diagnosis	One record per pathology report, per patient
**Imaging (prissmm_imaging)**	All imaging reports following cancer diagnosis	One record per imaging report, per patient
**Tumor markers^a^ (prissmm_tm)**	Serum-based tumor markers related to the cancer diagnosis/prognosis	One record per curated tumor marker result, per patient
**Medical oncologist assessments (prissmm_md)**	Select medical oncologist assessments following cancer diagnosis	One record per curated medical oncologist assessment, per patient
**Radiation therapy^a^ (ca_radtx)**	Radiation therapy corresponding to each cancer diagnosis	One record per radiation therapy and its associated cancer diagnosis, per patient
**Next-generation sequencing/cancer panel test (cpt)^b^**	Data on multi-gene panels that have been performed through next-generation sequencing assays	One record per cancer panel test and its associated cancer diagnosis, per patient
**Genomic data**		
**Mutations (mutations_extended)**	Mutation data corresponding to the next-generation sequencing report	One record for each mutation found through the targeted panel next-generation sequencing
**Fusions (fusions)**	Fusion data corresponding to the next-generation sequencing report	One record for each fusion found through the targeted panel next-generation sequencing
**Copy number alterations (cna)**	Copy number alteration data corresponding to the next-generation sequencing report	One record per gene and one column per sample

a For select cancer cohorts.

b The terms next-generation sequencing (NGS) and cancer panel test (CPT) are used interchangeably throughout this manuscript.

### 2.2 Select a cohort of patients for analysis: *create_analytic_cohort()*

The ***create_analytic_cohort()*** function can be used to subset the full GENIE BPC data release to a cohort of patients based on specified cancer diagnosis and/or cancer-directed drug regimen information. The following code demonstrates the selection of patients with a Stage IV adenocarcinoma NSCLC diagnosis treated with Carboplatin/Pemetrexed or Cisplatin/Pemetrexed with or without Bevacizumab as the first regimen following diagnosis. A summary of the cohort is optionally returned using the {gtsummary} package (Tables 3, 4, 5 and 6) (Sjoberg, *et al.*, 2021). The summary tables are stored with the prefix ‘tbl_’ as part of the list returned by ***create_analytic_cohort()*** and can be accessed from the *nsclc_stg_iv_adeno* list via: *nsclc_stg_iv_adeno$tbl_overall_summary, nsclc_stg_iv_adeno$tbl_cohort, nsclc_stg_iv_adeno$tbl_drugs and nsclc_stg_iv_adeno$tbl_ngs.*

**Table 3.** Summary of data returned from ***create_analytic_cohort()****(nsclc_stg_iv_adeno$tbl_overall_summary)*

Characteristics	*N* = 241 patients^a^
Number of diagnoses per patient in cohort_ca_dx data frame	
1	241 (100)
Number of regimens per patient in cohort_ca_drugs data frame	
1	241 (100)
Number of CPTs per patient in cohort_ngs data frame	
1	222 (92)
2	18 (7.5)
4	1 (0.4)

*Note*: The variables shown on Tables 3 are not output on any data frame returned by ***create_analytic_cohort()*** but are included in the summary tables to show the user the number of records per patient in each of the corresponding datasets.

a *n* (%).

**Table 4.** Summary of data returned in *cohort_ca_dx* data frame *(nsclc_stg_iv_adeno$tbl_cohort)*

Characteristics	*N *= 241 diagnoses^a^
Cohort (cohort)	
NSCLC	241 (100)
Institution (institution)	
DFCI	92 (38)
MSK	118 (49)
VICC	31 (13)
Stage at diagnosis (stage_dx)	
Stage IV	241 (100)
Histology (ca_hist_adeno_squamous)	
Adenocarcinoma	241 (100)

a *n* (%).

**Table 5.** Summary of data returned in *cohort_ca_drugs* data frame *(nsclc_stg_iv_adeno$tbl_drugs)*

Characteristics	*N* = 241 regimens^a^
Cohort (cohort)	
NSCLC	241 (100)
Institution (institution)	
DFCI	92 (38)
MSK	118 (49)
VICC	31 (13)
Drugs in regimen (regimen_drugs)	
Bevacizumab, Carboplatin, Pemetrexed Disodium	52 (22)
Bevacizumab, Cisplatin, Pemetrexed Disodium	27 (11)
Carboplatin, Pemetrexed Disodium	124 (52)
Cisplatin, Pemetrexed Disodium	38 (15)

a *n* (%).

**Table 6.** Summary of data returned in *cohort_ngs* data frame *(nsclc_stg_iv_adeno$tbl_ngs)*

Characteristics	*N* = 262 cancer panel tests^a^
Cohort (cohort)	
NSCLC	262 (100)
Institution (institution)	
DFCI	99 (38)
MSK	126 (48)
VICC	37 (14)
OncoTree code (cpt_oncotree_code)	
LCLC	1 (0.4)
LUAD	253 (97)
LUAS	1 (0.4)
LUSC	1 (0.4)
NSCLC	4 (1.5)
NSCLCPD	2 (0.8)
Sequence assay ID (cpt_seq_assay_id)	
DFCI-ONCOPANEL-1	1 (0.4)
DFCI-ONCOPANEL-2	57 (22)
DFCI-ONCOPANEL-3	41 (16)
MSK-IMPACT341	3 (1.1)
MSK-IMPACT410	61 (23)
MSK-IMPACT468	62 (24)
VICC-01-SOLIDTUMOR	26 (9.9)
VICC-01-T5A	1 (0.4)
VICC-01-T7	10 (3.8)

a *n* (%).

nsclc_stg_iv_adeno <- create_analytic_cohort(

data_synapse = nsclc_cohort$NSCLC_v2.0,

stage_dx = ‘Stage IV’,

histology = ‘Adenocarcinoma’,

regimen_drugs = c(‘Carboplatin, Pemetrexed Disod i um’,

‘Cisplatin, Pemetrexed Disodium’,

‘Bevacizumab, Carboplatin, Pemetrexed Disodium’,

‘Bevacizumab, Cisplatin, Pemetrexed Disodium’),

regimen_type = ‘Exact’,

regimen_order = 1,

regimen_order_type = ‘within cancer’,

return_summary = TRUE)

It is important to note that some of the parameters that can be specified in ***create_analytic_cohort()***may have missing data. In the above example, the cohort was built based on records having a documented histology of adenocarcinoma. However, the histology variable includes missing data and any record without histology recorded does not meet the criteria specified and is excluded. Users should carefully review patterns of missing data in the *data_synapse* object prior to specifying the inclusion criteria in ***create_analytic_cohort()***.

### 2.3 Select corresponding unique next-generation sequencing reports: *select_unique_ngs()*

After selecting the analytic cohort of interest, the next step is to select a next-generation sequencing (NGS) report for each patient. Because inclusion criteria for the GENIE BPC data involves undergoing NGS, each patient has at least one sequencing report. Occasionally, diagnoses may have multiple associated NGS reports based on repeated testing over time or following testing of a primary versus metastatic site (Table 3). The NGS dataset serves as the link between the clinical and genomic data, where the NGS dataset includes a record for each NGS report per cancer diagnosis, including the NGS sample ID that is used to link to the genomic data files. Therefore, merging data from the NGS report onto the analytic cohort returned from ***create_analytic_cohort()*** allows users to utilize all clinical and genomic data available. Details regarding the structure of each dataset and the list of key variables for merging across datasets can be found in the Analytic Data Guides that accompany each data release, as well as in the Clinical Data Structure vignette, which can be accessed via ***vignette(topic = ‘clinical_data_structure_vignette’, package = ‘genieBPC’)***.

The ***select_unique_ngs()*** function may be used to select a unique NGS report for each diagnosis for the purpose of creating an analytic dataset based on user-defined criterion, including OncoTree code, primary versus metastatic tumor sample, and earliest versus most recent sequencing report (Kundra, *et al.*, 2021). If multiple reports for a patient remain available after applying the user-defined specifications, or if no specifications are provided, the panel with the largest number of genes will be selected by default. Sample selection is performed in the order that the arguments are specified in the function, regardless of the arguments’ order provided by the user; namely the OncoTree code is prioritized first, sample type is prioritized second, and finally the time is prioritized last. For patients with exactly one genomic sample, that unique genomic sample will be returned regardless of whether it meets the user-specified parameters. Running the ***select_unique_ngs()*** function will ensure that the resulting dataset returned by merging the next-generation sequencing report data onto the analytic dataset returned by ***create_analytic_cohort()*** will maintain a structure of one record per patient.

The following code selects one unique NGS report per patient in the *nsclc_stg_iv_adeno* analytic dataset.

nsclc_adeno_stg_iv_unique_sample <-

select_unique_ngs (

  data_cohort = nsclc_stg_iv_adeno$cohort_ngs,

  oncotree_code = ‘LUAD’,

  sample_type = ‘Metastasis’

)

The purpose of selecting a single NGS report per patient is to have unique genomic information available for each diagnosis. Alternatively, as opposed to selecting a single NGS report per patient, the analyst may instead be interested in investigating the genomic alteration status across NGS reports, when multiple reports are available. For example, if a patient has any NGS report that indicates an Epidermal Growth Factor Receptor (EGFR) mutation, that diagnosis could be classified as having an EGFR alteration. If this is the case, the user would replace Step 2.3 of this case study with an analysis step consolidating information across multiple NGS reports, when they are available.

### 2.4 Visualize drug regimen sequences in an interactive sunburst plot: *drug_regimen_sunburst()*

**Fig. 2.**
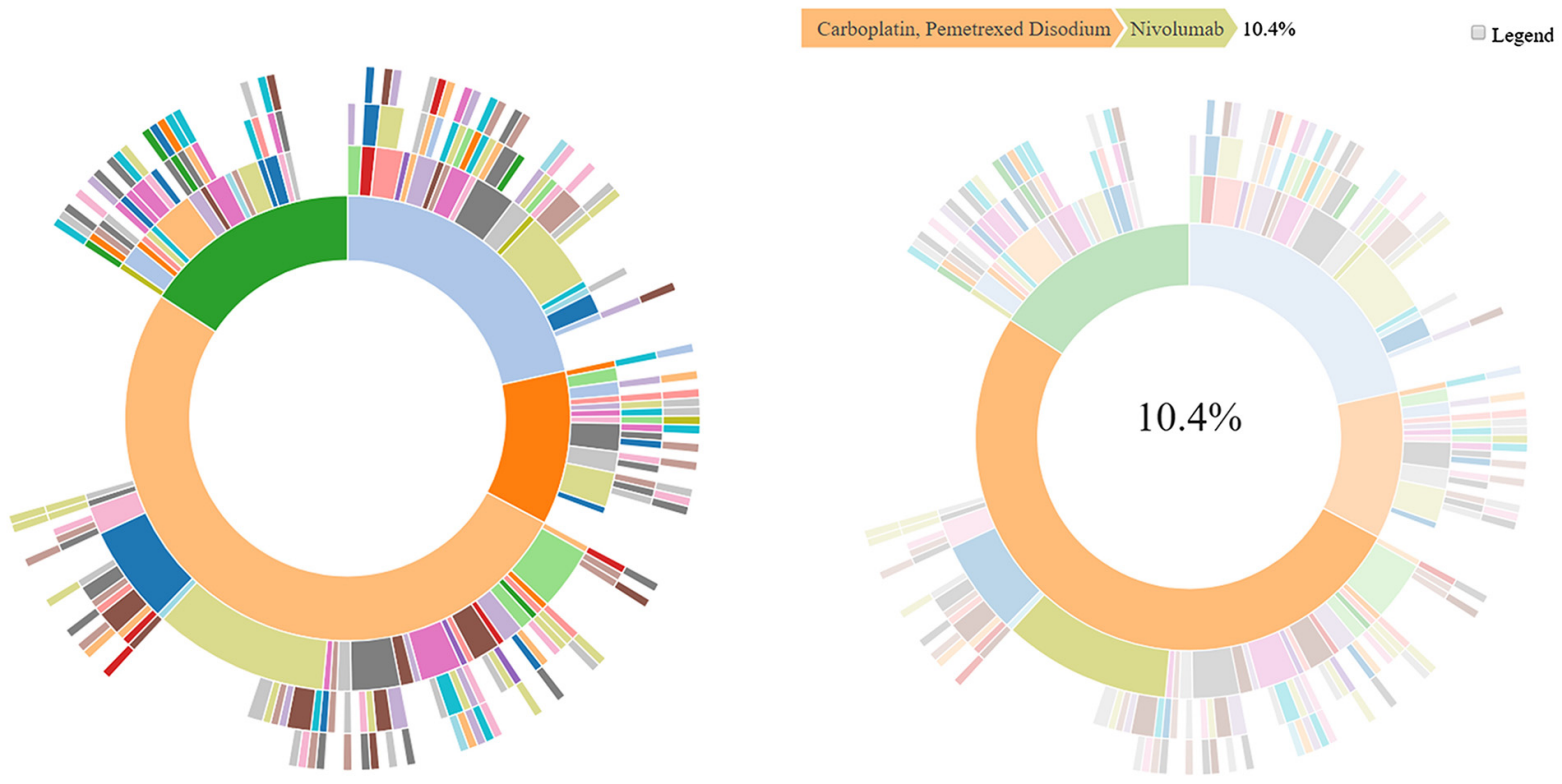
Example of an interactive sunburst plot showing the first four drug regimens received (left panel) and highlighting a select treatment regimen sequence (right panel)

To visualize the treatment patterns for the diagnoses of interest, a user can create an interactive sunburst plot to show the proportion of patients with each drug regimen trajectory. Each ring of the sunburst corresponds to a regimen, such that the innermost ring is the first regimen, the second innermost ring is the second regimen, and so on. To create a sunburst plot of cancer-directed drug regimen data, the user must specify the lists returned from ***pull_data_synapse()*** and ***create_analytic_cohort()*** and can optionally specify the maximum number of regimens to display using the ***drug_regimen_sunburst()***function. The below call to ***drug_regimen_sunburst()*** returns the object *sunplot$sunburst_plot* (Fig. 2).

sunplot <- drug_regimen_sunburst(

data_synapse = nsclc_cohort$NSCLC_v2.0,

data_cohort = nsclc_stg_iv_adeno,

max_n_regimens = 4)

When the user generates the sunburst plot, all colors are shown with the same hue as in the left panel. When the user hovers over a specific part of the figure, the colors corresponding to any other treatment pattern are faded so that the treatment pattern of interest is more clearly distinguished, as shown in the right panel, which displays the same sunburst plot as in the left panel but as it is shown when the user hovers over the most common first-line regimen (Carboplatin, Pemetrexed Disodium in orange) followed by the most common second-line regimen (Nivolumab in green).

Hovering over different bands within the interactive sunburst plot will show the treatment regimen pattern and the percentage of patients that received that regimen for that order of therapy. In this case study, the sunburst shows that among patients who received Carboplatin/Pemetrexed as their first regimen, the largest percentage went on to receive Nivolumab as their second regimen (Fig. 2).

The ***drug_regimen_sunburst()*** function is a wrapper function for ***sunburstR::sunburst()***, and accepts additional parameters available in the ***sunburstR::sunburst()*** function that modify the presentation of the sunburst, including the color scheme (Bostock *et al.*, 2021).

## 3 Discussion

The GENIE BPC data provide a resource that can be used to advance precision medicine in oncology by investigating the associations between genomic features and clinical outcomes. With endpoints that are not typically recorded in structured fields within EHR, these data are uniquely rich, capturing treatment trajectories alongside genomic characterization and salient outcomes in oncology. The {genieBPC} R package is the first set of tools created to facilitate accessing and processing GENIE BPC data to create analytic cohorts that are ready for clinico-genomic modeling and analyses. This streamlined process is particularly important given that updates to the data are periodically released; these tools facilitate updating previous analyses as new cancer cohorts and new versions of the data continue to become available.

Though the {genieBPC} package facilitates building an analytic cohort, it is up to the discretion of the analyst to develop an appropriate statistical analysis plan. Because of the variety of research questions that can be answered with these data and the analytic decisions required, the package does not provide an analytic framework but is focused on providing a concise way to build a cancer cohort by a cancer diagnosis and/or treatment information. Additionally, as is the case with most datasets, there are missing values in the GENIE BPC data releases. Because there are several options for handling missing data and any missing data should be thoroughly investigated and accounted for in the context of a specific research study, the {genieBPC} package does not impute or otherwise manipulate missing data. As with all observational datasets, the GENIE BPC data are subject to biases, which should be adequately evaluated and handled appropriately in the analysis phase (Brown *et al.*, 2021).

Future work on this package includes the addition of cohort selection criteria based on site(s) of metastasis at diagnosis (Stage IV) or following diagnosis (Stages I–III), as well as the option to select drugs based on drug classes, such as immunotherapy, targeted therapy and chemotherapy.

## 4 Conclusion

The {genieBPC} R package provides a streamlined approach to creating structured analytic datasets that will facilitate reproducible analysis of the real-world, clinico-genomic data arising from Project GENIE BPC. As the data for the first two cancer cohorts (non-small cell lung cancer and colorectal cancer) were publicly released in 2022, and with additional cancer cohorts to be publicly released in the coming year, this R package presents a timely solution for processing a complex collection of clinical and genomic data elements.
